# Outcomes from an infectious disease physician-guided evaluation of hospitalized persons under investigation for coronavirus disease 2019 (COVID-19) at a large US academic medical center

**DOI:** 10.1017/ice.2020.434

**Published:** 2020-08-24

**Authors:** Caitlin M. Dugdale, Sarah E. Turbett, Suzanne M. McCluskey, Kimon C. Zachary, Erica S. Shenoy, Andrea L. Ciaranello, Rochelle P. Walensky, Eric S. Rosenberg, Melis N. Anahtar, David C. Hooper, Emily P. Hyle

**Affiliations:** 1 Medical Practice Evaluation Center, Department of Medicine, Massachusetts General Hospital, Boston, Massachusetts; 2 Division of Infectious Diseases, Department of Medicine, Massachusetts General Hospital, Boston, Massachusetts; 3 Harvard Medical School, Boston, Massachusetts; 4 Department of Pathology, Massachusetts General Hospital, Boston, Massachusetts; 5 Infection Control Unit, Massachusetts General Hospital, Boston, Massachusetts

## Abstract

We describe an approach to the evaluation and isolation of hospitalized persons under investigation (PUIs) for coronavirus disease 2019 (COVID-19) at a large US academic medical center. Only a small proportion (2.9%) of PUIs with 1 or more repeated severe acute respiratory coronavirus virus 2 (SARS-CoV-2) nucleic acid amplification tests (NAATs) after a negative NAAT were diagnosed with COVID-19.

Prompt isolation and diagnosis of hospitalized patients with coronavirus disease 2019 (COVID-19) is essential for the prevention of nosocomial transmission and allocation of personal protective equipment (PPE). The mainstay of COVID-19 diagnosis is detection of severe acute respiratory coronavirus virus 2 (SARS-CoV-2) RNA through nucleic acid amplification tests (NAATs). However, NAAT sensitivity varies by specimen quality, timing, and severity of infection.^1,2^ The sensitivity of a single NAAT is ~70%, while repeated testing increases sensitivity to 88%.^2^ Lower respiratory tract (LRT) specimens generally have higher sensitivity than upper respiratory tract (URT) specimens.^2^

Based on these data, the Infectious Diseases Society of America (IDSA) recommends repeating a NAAT, preferably with an LRT specimen, when clinical suspicion for COVID-19 remains moderate or high.^2^ We describe an approach to the evaluation and isolation of hospitalized persons under investigation (PUIs) for COVID-19 in whom clinical evaluation and additional diagnostic testing after a first negative NAAT were guided by infectious disease (ID) physician review.

## Methods

We performed a retrospective analysis of adult COVID-19 PUIs who were hospitalized at Massachusetts General Hospital for ≥24 hours from March 23 to May 18, 2020, using Epic reporting workbench data. We included patients with symptoms consistent with COVID-19 symptoms (eg, cough, shortness of breath, fever, chills, myalgias, sore throat, headaches, anosmia, or ageusia) or without COVID-19 symptoms but at elevated risk for COVID-19 (eg, from a skilled nursing facility, undomiciled, or recently in close contact with someone with COVID-19), who underwent evaluation with ≥1 SARS-CoV-2 NAATs. We analyzed both types of PUIs together.

All patients included in this study had a NAAT within 24 hours of admission and were isolated following droplet, contact, and eye protection precautions.^3^ We excluded patients whose first NAAT was performed as an outpatient or >24 hours after admission or who remained COVID-19 PUIs at the time of death or discharge. All SARS-CoV-2 NAATs were performed using Food and Drug Administration (FDA) emergency use authorized (EUA) assays. The test turnaround time for inpatient URT NAATs ranged from 1 to 30 hours and the test turnaround time for LRT NAATs ranged from 1 to 9 days.

Throughout the study period, ID faculty and senior fellows evaluated the clinical, epidemiologic, laboratory, and radiographic parameters for each COVID-19 PUI daily by remote chart review and communication with frontline providers. ID physicians provided guidance on removal of isolation precautions if COVID-19 was deemed unlikely, or recommendations for additional diagnostics if clinical suspicion for COVID-19 was moderate-high. ID physicians reviewed 80–110 PUIs per day from 6 a.m. to midnight, requiring ~70 person hours per day. Challenging cases for which the physician sought further infection control team input were discussed on thrice-daily rounds to establish consensus. We characterized the frequency and yield of repeated NAATs, utilization of chest computed tomography (CT) among COVID-19 PUIs, and the time from first NAAT until discontinuation of isolation. The study was approved by the Mass General Brigham Institutional Review Board.

## Results

**Fig. 1.**
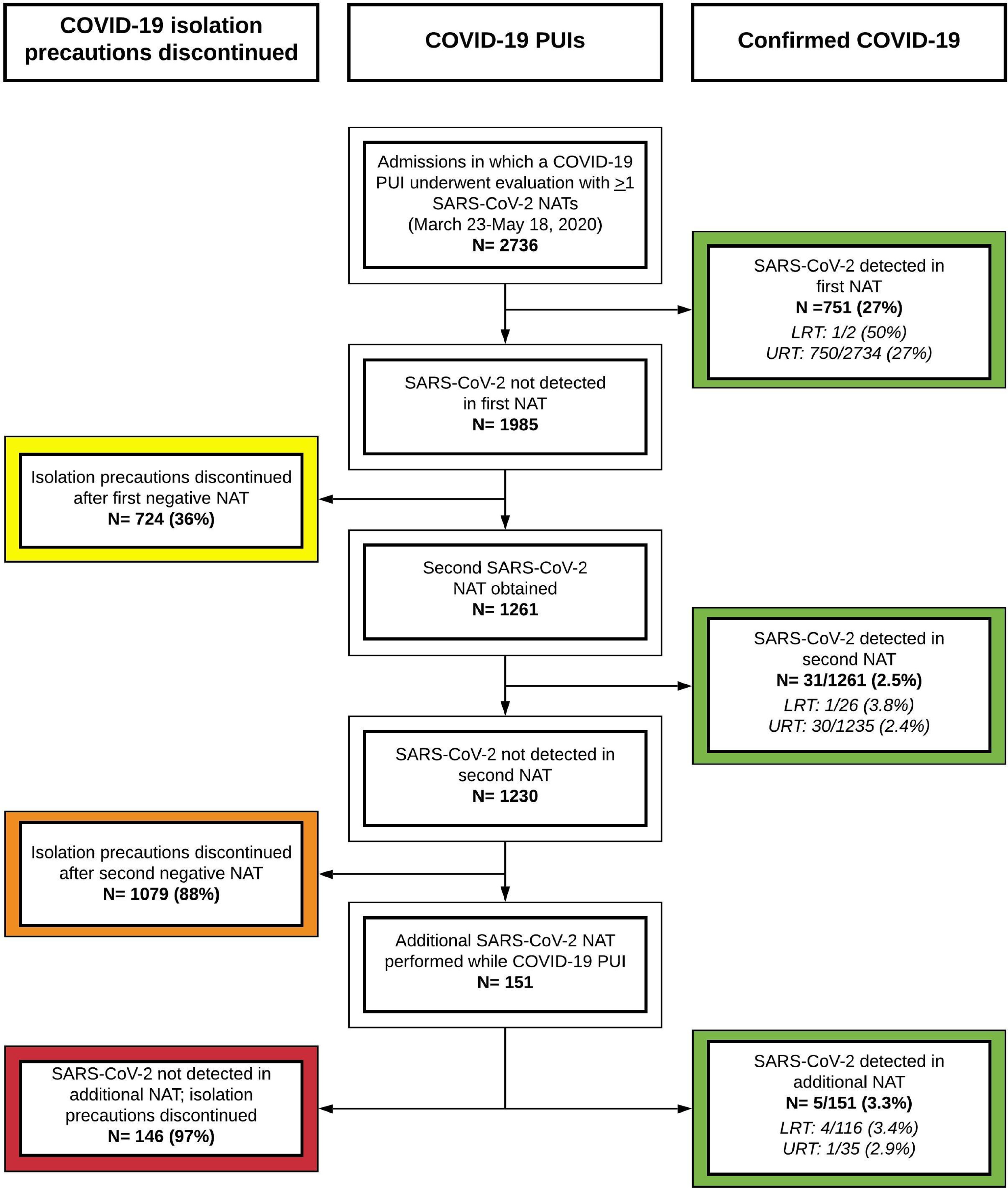
Flow diagram of hospitalized COVID-19 PUIs with SARS-CoV-2 testing at Massachusetts General Hospital. All hospitalized patients with symptoms concerning for COVID-19 were eligible for testing with a SARS-CoV-2 NAAT. Patients with a positive NAAT were diagnosed with COVID-19 (green). COVID-19 PUIs for whom the ID physicians had low clinical suspicion for COVID-19 had COVID-19 isolation precautions discontinued after 1 negative NAAT (yellow). COVID-19 PUIs for whom the ID physicians had moderate-high clinical suspicion for COVID-19 had additional workup, including 1 or more additional NAATs. Among COVID-19 PUIs without any positive NAAT, most had isolation precautions discontinued after 2 negative NAATs (orange), but a substantial minority remained COVID-19 PUIs and underwent additional NAATs (red) given ongoing clinical concern for COVID-19 infection. Note. PUI, person under investigation; SARS-CoV-2, severe acute respiratory syndrome coronavirus 2; NAAT, nucleic acid amplification test; LRT, lower respiratory tract; URT, upper respiratory tract.

In total, COVID-19 PUIs from 2,736 unique hospitalizations underwent evaluation using ≥1 SARS-CoV-2 NAATs (Fig. 1). Of these, 751 patients (27%) had SARS-CoV-2 detected by the first NAAT. We considered 724 (36%) of the 1,985 first negative NAATs to be true negatives based on ID physician review. Of the 1,261 remaining COVID-19 PUIs, 31 (2.5%) had SARS-CoV-2 detected by a second NAAT. Among 1,230 patients with 2 negative NAATs, 151 (12%) had additional NAATs performed, of whom 5 (3.3%) had detectable SARS-CoV-2 RNA. Only 142 LRT specimens were obtained among patients with a first negative NAAT, of which 5 (3.5%) were positive. In total, 36 of 1,261 patients (2.9%) with repeated testing were diagnosed with COVID-19 after a first negative NAAT.

Diagnostic imaging was performed in a subset of patients to inform ongoing suspicion for COVID-19. Chest CTs were obtained in 92 patients (12%) who remained in isolation <24 hours; in 315 patients (28%) who remained in isolation 24–96 hours, and in 47 patients (48%) who remained in isolation at >96 hours. Chest CT utilization increased among PUIs for whom more NAATs were performed. Among all PUIs, 741 (38%) had isolation discontinued within 24 hours of the first NAAT, while 98 (5%) remained in isolation for >96 hours due to ongoing suspicion for COVID-19 (Table 1).

**Table 1.** Duration of Isolation Among Hospitalized Patients Without COVID-19 Infection by Number of SARS-CoV-2 NAATs and Chest CT scans Performed

No. of Tests	Duration of COVID-19 Isolation, No. (%)		
	<24 Hours	24–96 Hours	>96 Hours
Any NAATs (N=1,949)	741	1,110	98
Chest CT	92 (12)	315 (28)	47 (48)
Max 1 NAAT (N=724)	552	167	5
Chest CT	67 (12)	27 (16)	2 (40)
Max 2 NAATs (N=1,079)	186	860	33
Chest CT	25 (13)	262 (30)	17 (52)
≥3 NAATs (N=146)	3	83	60
Chest CT	0 (0)	26 (31)	28 (47)

Note. NAAT, nucleic acid amplification test; CT, computed tomography.

Two patients had a subsequent positive NAAT within 7 days of discontinuing isolation without evidence of onward nosocomial transmission. The first patient was admitted from a skilled nursing facility with confirmed cases of COVID-19. She was asymptomatic during her initial NAAT, which was likely obtained within the incubation period. Development of a cough 6 days later triggered repeated testing that was positive. The second patient had 2 negative URT NAATs performed within 24 hours of symptom onset. A URT NAAT repeated 5 days later prompted by a worsening cough returned a positive result with low cycle thresholds (ORF1ab Ct, 16.2 and E gene Ct, 16.6), most consistent with early infection.

## Discussion

We used a systematic evaluation of hospitalized COVID-19 PUIs by ID physician review to achieve accurate COVID-19 diagnoses, to minimize nosocomial transmission, and to conserve PPE. This approach was resource intensive but effective. Subsequent diagnosis of COVID-19 occurred in only 2 of 1,949 patients (0.10%) after initial evaluation prompted resolution of PUI status and cessation of isolation.

Although IDSA guidelines recommend repeated NAATs among symptomatic inpatients, we diagnosed COVID-19 in only 2.9% of all PUIs with repeated NAATs despite the high prevalence of infection. This low false-negative rate may be due to a smaller incremental diagnostic yield of repeated testing among hospitalized patients who may present later in disease when NAATs are less sensitive, or due to variation in specimen quality.^1,2^ Importantly, only 11% of COVID-19 PUIs with repeated NAATs had LRT performed due to limited access, long turnaround times, and inability to produce sputum. Given the higher sensitivity of LRT testing, validation of LRT specimens on FDA EUA SARS-CoV-2 NAAT platforms should be prioritized to improve access to this testing modality.^2^

Improved understanding of transmission dynamics is critical to guide recommendations regarding optimal testing approaches and duration of isolation for COVID-19 PUIs.^4^ While prolonged detection of SARS-CoV-2 RNA is well described, it is unlikely that the virus remains transmissible throughout that duration.^3^ In a study examining 90 positive SARS-CoV-2 NAATs, live virus was not isolated beyond 8 days after symptom onset.^3,5^ However, in a preprint study of 129 patients with severe COVID-19 disease, live virus was cultured from a patient at 20 days after symptom onset.^6^ In both studies, low viral load was a strong predictor of culture negativity.^5,6^ If transmissions do not occur from patients with false negative NAATs, then resource-intensive evaluations with ID review could be curtailed.

This study had several limitations. First, no gold standard has been established for COVID-19 diagnosis. Despite efforts to prevent missed diagnoses, isolation precautions may have been discontinued in patients with undiagnosed infection. This approach to diagnosis of COVID-19 PUIs required >5,000 ID physician person hours over 2 months, which is not feasible in many settings. Exclusion of hospitalized patients initially tested as outpatients may underestimate the yield of repeated NAATs. These data also predate widespread availability of SARS-CoV-2 serology and, therefore, do not capture its diagnostic impact.^7^

We demonstrated the effectiveness of a detailed clinical review process with a low rate of observed missed COVID-19 diagnoses, but this approach is highly resource intensive. Improved diagnostic tests that are sensitive and specific throughout illness, validated algorithms to evaluate PUIs, and an improved understanding of SARS-CoV-2 transmissibility are essential to guide more efficient approaches to COVID-19 diagnosis and management of isolation precautions.
